# Expression and Purification of Integral Membrane Fatty Acid Desaturases

**DOI:** 10.1371/journal.pone.0058139

**Published:** 2013-03-08

**Authors:** Haiqin Chen, Zhennan Gu, Hao Zhang, Mingxuan Wang, Wei Chen, W. Todd Lowther, Yong Q. Chen

**Affiliations:** 1 State Key Laboratory of Food Science and Technology, School of Food Science and Technology, Jiangnan University, Wuxi, Jiangsu Province, People’s Republic of China; 2 Department of Cancer Biology, Wake Forest School of Medicine, Winston-Salem, North Carolina, United States of America; 3 Department of Biochemistry, Wake Forest School of Medicine, Winston-Salem, North Carolina, United States of America; Max Delbrueck Center for Molecular Medicine, Germany

## Abstract

Fatty acid desaturase enzymes perform dehydrogenation reactions leading to the insertion of double bonds in fatty acids, and are divided into soluble and integral membrane classes. Crystal structures of soluble desaturases are available; however, membrane desaturases have defied decades of efforts due largely to the difficulty of generating recombinant desaturase proteins for crystallographic analysis. *Mortierella alpina* is an oleaginous fungus which possesses eight membrane desaturases involved in the synthesis of saturated, monounsaturated and polyunsaturated fatty acids. Here, we describe the successful expression, purification and enzymatic assay of three *M. alpina* desaturases (FADS15, FADS12, and FADS9-I). Estimated yields of desaturases with purity >95% are approximately 3.5% (Ca. 4.6 mg/L of culture) for FADS15, 2.3% (Ca. 2.5 mg/L of culture) for FADS12 and 10.7% (Ca. 37.5 mg/L of culture) for FADS9-I. Successful expression of high amounts of recombinant proteins represents a critical step towards the structural elucidation of membrane fatty acid desaturases.

## Introduction

Lipids are first synthesized as saturated fatty acids, and double bonds are introduced post-synthetically by oxygen-dependent enzymes known as fatty acid desaturases, in a process that is initiated by abstraction of hydrogen from a methylene group. Fatty acid desaturases are divided into soluble and integral membrane classes, which may have evolved independently [1]. The acyl-ACP desaturases are soluble enzymes found in the plastids of higher plants, whereas the more widespread class of integral membrane acyl-CoA desaturases is found in endomembrane systems in prokaryotes and eukaryotes [2]. Fatty acid desaturases in each class are closely related homologs based on their amino acid sequences, and yet perform highly regio- and stereo-selective reactions on long-chain fatty acids composed of essentially equivalent methylene chains that lack distinguishing landmarks close to the site of desaturation. As pointed out by Nobel Laureate Dr. Konrad Bloch, this region- and stereo-specific removal of hydrogen “would seem to approach the limits of the discriminatory power of enzymes” [3].

The membrane class of desaturases consists of enzymes with c5, c6, c9, c12 or ω3 regio-selectivity. Structure determination would significantly improve our understanding of the structure-function relationships of this diverse class of proteins; however, there has been little progress, despite decades of efforts, due largely to the difficulty of generating recombinant membrane desaturase proteins for crystallographic analysis. Mammalian cells possess c5, c6 and c9, but lack c12 and ω3 desaturases [4], [5]. *Mortierella alpina* belongs to the subphylum of *Mucoromycotina* [6]. It is an oleaginous fungus that can produce lipids up to 50% of its dry weight. We have recently characterized the *M. alpina* genome [7] which encodes one c5, two c6, three c9, one c12 and one ω3 desaturase (**Figure S1**). Therefore, *M. alpina* has all known regio-selective groups of membrane desaturases.

Membrane desaturases have been expressed in various hosts, such as *Escherichia coli, Saccharomyces cerevisiae, Aspergillus oryzae, Mortierella alpina* and cell-free system, for biochemical characterizations [8], [9], [10], [11], [12], [13], [14], [15], [16]. However, none of these expression systems could achieve sufficient amount of desaturase proteins for crystal structure analysis. In the present study, we expressed *M. alpina* c9, c12 and ω3 desaturases (*FADS9-I*, *FADS12* and *FADS15*) in the methylotrophic yeast *Pichia pastoris*, purified the recombinant proteins and determined their enzymatic activities. High yield of these proteins paves the way for structural characterization of membrane desaturases.

## Materials and Methods

### Plasmids, Strains and Growth Conditions

Plasmids were amplified in DH5α bacteria grown in Luria-Bertani (LB) medium containing 100 µg/mL of ampicillin at 37°C with 250 rpm shaking, and purified using Qiagen Maxi Kit (Qiagen, CA). The expression vector pPinkα-HC was purchased from Invitrogen (Carlsbad, CA). The *P. pastoris* strains used for protein expression were PichiaPink strain 1 (*ade2*), strain 2 (*ade2, pep4*), strain 3 (*ade2, prb1*) and strain 4 (*ade2, pep4, prb1*), purchased from Invitrogen. *P. pastoris* strains were cultured in Yeast Extract Peptone Dextrose (YPD, PichiaPink media kit Cat# A11156, Invitrogen) at 28°C with 250 rpm shaking.

*Mortierella alpina* (#32222, American Type Culture Collection, Manassas, Virginia, USA) was cultured as described previously [7].

### Expression Vector Construction

*M. alpina* RNA extraction was performed using Trizol Reagent (Invitrogen, CA) according to the manufacturer’s instructions. Total RNA was reverse transcribed with SuperScript® III First-Strand Synthesis SuperMix (Invitrogen) following the manufacturer’s instructions. Using both C- and N-terminal sequences as primers (**Table S1**), desaturase coding sequences were PCR amplified as follows: denaturation at 95°C for 30 sec, annealing at 55°C for 45 sec and extension at 72°C for 1 min for 35 cycles. The amplified products were cloned into a modified pET19 vector (Novagen) derivative containing a PreScission protease cleavage site (GE Healthcare) between the multiple cloning site and N-terminal His tag ([17] to construct pET19b-FADS15, pET19b-FADS12 and pET19b-FADS9-I). The desaturase genes, including the His-Tag and PreScission protease cleavage site, were then PCR amplified using primers SF1 and SR1-SR3 (**Table S1**). The PCR conditions used were the same as the first step for cDNAs. The PCR fragments were then purified and inserted into pPinkα-HC to generate the expression vectors pPinkα-HC-FADS15, pPinkα-HC-FADS12 and pPinkα-HC-FADS9-I. The presence of the inserts in the plasmids was confirmed by restriction digestion analysis and sequencing. The strategy used for constructing desaturase expression vectors is shown in **Figure S2**.

### PichiaPink Transformation

Desaturase expression vectors and pPinkα-HC (negative control vector) were linearized with restriction enzyme *Spe* I and transformed into *P. pastoris* strains (PichiaPink strain 1, 2, 3 and 4) using the MicroPulser Electroporator (Bio-Rad Laboratories, Hercules, CA) according to the User Manual of PichiaPink Expression System (Invitrogen). *P. pastoris* were incubated with YPDS media (YPD with 1 M sorbitol) in the Gene Pulser Cuvettes at 28°C for 2 hr without shaking, spread onto PAD (Pichia Adenine Dropout) agar selection plates, and then incubated at 28°C for 4 days until distinct colonies were formed. Eight white colonies for each transformation were picked and plasmid integration in the yeast genome was confirmed by PCR.

Isolated clones were individually inoculated into 10 mL of BMGY medium (Buffered Glycerol-complex Medium, 1% yeast extract; 2% peptone; 100 mM potassium phosphate, pH 6.0; 1.34% YNB-Yeast Nitrogen Base; 0.0004% biotin; 1% glycerol) in 50 mL conical tubes. The cells were grown for 48 hr at 28°C with vigorous shaking at 250 rpm. Then, the cultures were centrifuged at 1,500 g for 5 min at room temperature, the cell pellets were resuspended in 2 mL of BMMY medium (Buffered Methanol-complex Medium, 1% yeast extract; 2% peptone; 100 mM potassium phosphate, pH 6.0; 1.34% YNB; 0.0004% biotin; 0.5% methanol) and cultured at 28°C with shaking at 250 rpm to induce the expression. After continuous cultivation for 72 hr with daily addition of 0.5% methanol, cells were harvested by centrifuging for 10 min at 1500 g. Supernatant was transferred to a separate tube and both the supernatant and cell pellet were stored at −80°C until ready for assay. Supernatants and cell pellets were analyzed for protein expression by SDS-PAGE Coomassie blue staining and Western blot.

### Optimized Protein Expression Condition

Individual colonies of *P. pastoris*-FADS15, FADS12 and FADS9-I were inoculated into 10 mL of BMGY medium in 50 mL conical tubes and cultured for 48 hr at 28°C at shaking speed of 250 rpm. Then, 2.5 mL of culture were inoculated into 50 mL of BMGY medium in 250-mL volume shaker flasks and grown at 28°C for 24 hr at 250 rpm. The cells were collected by centrifugation at 1500 g for 10 min, and resuspended in 10 mL induction medium (BMMY medium with 0.5% methanol) in a 100-mL shaker flask. The induction of protein expression was performed for 96 hr at 28°C with 250 rpm agitation and daily addition of 0.5% methanol. Samples were collected at 0, 6, 24, 48, 72 and 96 hr for measuring cell density at OD_600_, wet cell weight and total protein concentration, and for Western blot analysis of desaturase expression levels.

### Protein Analyses

The cell pellets and supernatants were collected by centrifuging 100 µL cell culture at 1500 g for 10 min. Cell pellets were resuspended in 100 µL lysis buffer (20 mM Tris.Cl pH7.9, 1 mM EDTA, 5% Glycerol) with an equal volume of 0.5 mm Glass Beads (Biospec products, Inc.), and vortexed for 10 min at 4°C. Cell lysates were mixed with 4×SDS sample buffer and heated for 5 min at 95°C. About 5 µl sample was loaded onto Mini-Protein Precast Gels (4–15%, Bio-Rad Laboratories, Cat #456–1086), and ran for 40 min at 150 V. Then, the SDS-PAGE gels were used for Coomassie blue stain, Invision His-Tag in-gel stain (Invitrogen) or Western blot.

For Western blot analysis, protein gels were transferred onto a nitrocellulose transfer membrane (Schleicher & Schuell GmbH, Germany) by electroblotting (100 V, 2 hr) using Mini Trans-Blot electrophoretic transfer cell (Bio-Rad Laboratories). The membrane was blocked with 3% BSA in TBST (150 mM NaCl, 10 mM Tris-Cl pH 7.5, 0.05% Tween20), and probed with mouse Penta·His antibody (Invitrogen) followed by HRP-conjugated goat anti-mouse IgG (GE Healthcare). Blots were then incubated with enhanced chemiluminescence reagent (ECL, GE healthcare) and analyzed using Fluorchem E (Cell Biosciences, Inc.).

The total protein concentration was determined with Pierce BCA protein assay kit (Thermo Scientific). The quantification of target protein on Coomassie blue stained gel was performed using known concentrations of BSA as standard, and analyzed with the AlphaView SA software (Cell Biosciences, Inc.).

### Cell Fractionation

All purification procedures were performed at 4°C. Cells harvested from 800 µL of culture were suspended in 800 µL of lysis buffer. After addition of 0.5 mm glass beads to the cell suspension, *P. pastoris* cells were disrupted by vortexing at 4°C for 10 min. Cell lysis efficiency was usually more than 95% evaluated using a light microscope. Intact cells and cell debris were removed from the membrane suspension by low speed centrifugation (500 g, 10 min at 4°C). Then various centrifugation speeds and time (1,000 g for 10 min; 10,000 g for 10 min; 10,000 g for 20 min; 20,000 g for 10 min; 20,000 g for 20 min) were used to determine the best centrifugation conditions for collecting the membrane fraction.

### Protein Solubilization

Fractions containing recombinant desaturases were solubilized in buffer, containing 20 mM Tris.Cl, pH 7.9, 500 mM NaCl, 10% glycerol, 0.1 mM EDTA, and different concentrations (0.5%, 1%, 2%) of various detergents (Tween 20, Tween 80, Nonidet P-40, DDM, Fos-Choline 12, Fos-Choline 16) at 4°C for different times (0.5, 1, 1.5, 2 hr and overnight). The insoluble materials were removed by centrifugation at 25,000 g for 30 min at 4°C.

### Protein Affinity Purification

Optimized culture and protein solubilization conditions were used for the subsequent purification process. His Mag Sepharose™ Ni affinity beads (GE Healthcare) were washed with binding buffer (20 mM Tris.Cl, pH 7.9, 500 mM NaCl, 10% glycerol, 0.1 mM EDTA, 0.5% Fos-Choline 16, 5 or 20 mM imidazole) and added to the solubilized fractions after detergent incubation. The bead-protein sample mixtures were incubated for 45 min at 4°C with end-over-end mixing. After washing three times with binding buffer containing 5 mM or 20 mM imidazole, desaturase enzymes were eluted with elution buffer (20 mM Tris.Cl, pH 7.9, 500 mM NaCl, 10% glycerol, 0.1 mM EDTA, 0.5% Fos-Choline 16, 500 mM imidazole). The purified FADS15, FADS12 and FADS9-I proteins were stored at −80°C in aliquots. The quantity and quality of these purified enzymes were analyzed by SDS-PAGE, mass spectrometry and desaturase activity assay. Protein purity was presented in percentage, dividing the amount of a given desaturase protein quantified on gel by the amount of loaded protein quantified by the BCA protein assay.

### Fatty Acid Analysis

Approximately 20 mg of *P. pastoris* cell pellets were collected and used for each lipid extraction with the method of Bligh and Dyer [18] under acidified conditions with pentadecanoic acid and heneicosanoic acid added as internal standards. The solvent from the extract was removed under a stream of nitrogen. Lipids were saponified in 1 mL of freshly prepared 5% ethanolic potassium hydroxide at 60°C for 1 hr under an argon atmosphere. After cooling, 1 mL of water was added to the samples and non-saponifiable lipids were extracted into 3 mL of hexane. The aqueous layer was acidified with 220 µL of 6 M hydrochloric acid and the fatty acids extracted into 3 mL of hexane. After removing the hexane in a stream of nitrogen, fatty acids were converted to methyl esters by first treating with 1 mL of 0.5 M methanolic sodium hydroxide at 100°C for 5 min under argon followed by 1 mL of 14% methanolic boron trifluoride at 100°C for 5 min under argon [19]. After cooling, the sample was mixed with 2 mL of hexane followed by 4 mL of saturated aqueous sodium chloride. After separating the phases, aliquots of the hexane layers were diluted 24-fold with hexane and then analyzed by GC/MS. One µL was injected in the splitless mode onto a 30 m×250 µm DB-WAXETR column (Agilent Technologies, Santa Clara, California) with 0.25 µm film thickness. The temperature program was as follows: 100°C for 2 min, ramp to 200°C at 16°C per min, hold for one min, ramp to 220°C at 4°C per min, hold one min, ramp to 260°C at 10°C per min, and hold for 11 min. Helium was the carrier gas at a constant flow of 1.5 mL/min. The mass spectrometer was operated in positive-ion electron impact mode with interface temperature 260°C, source temperature 200°C, and filament emission 250 µA. Spectra were acquired from m/z 50 to 450 with a scan time of 0.433 s. Lower-boiling fatty acid methyl esters were quantified using the pentadecanoic acid internal standard, whereas higher-boiling methyl esters were quantified using the heneicosanoic acid internal standard.

### In vivo Desaturase Activity Analysis

Individual colonies of *P. pastoris*-FADS15, FADS12 and FADS9-I were cultured as described in the Recombinant protein expression section. Protein expression was induced for 72 hr with 0.5% methanol. Cell pellets were collected by centrifugation and stored at −80°C for fatty acid analysis.

### In vitro Desaturase Activity Analysis

20 µL of the purified protein was added to 200 µL of yeast EGY49 cell homogenate, prepared by breaking cells with 0.5 mm glass beads in lysis buffer (20 mM Tris-HCl pH7.9, 1 mM EDTA, 5% Glycerol). The enzyme reactions were performed at 28°C for 3 h with shaking (250 rpm), and the assay mixture (220 µL) were stored at −80°C for fatty acid analysis.

## Results

### Sequence of M. alpina Fatty Acid Desaturases

Recently, we sequenced the genome and EST of *M. alpina* ATCC #32222. The cDNA and protein sequences of FADS9-I, FADS12 and FADS15 were compared to published sequences from other strains of *M. alpina* (**Figure S3-S5**). The FADS12 and FADS9-I genes from *M. alpina* ATCC#32222 are 99.9% and 98.4% identical, respectively, to the corresponding genes from *M. alpina* 1s-4. The FADS12 and FADS9-I proteins from *M. alpina* ATCC#32222 are 100% and 99.6% identical, respectively, to these proteins from *M. alpina* 1s-4. The high similarity of FADS12 and FADS9-I genes between two strains indicates that these genes are highly conserved in *M. alpina*. Interestingly, the FADS15 gene is much less conserved at both DNA (93.1% identity) and protein (97.9%) levels.

### Expression of Fatty Acid Desaturases in the PichiaPink System

After several unsuccessful attempts to express recombinant *M. alpina* desaturases in bacteria, we tried to express FADS15, FADS12 and FADS9-I in the *Pichia pastoris* PichiaPink expression system (Invitrogen). Our data showed that PichiaPink strain 2(ade2, pep4) supported the highest level of expression for FADS15, 12 and 9-I. Interestingly, all recombinant desaturase proteins remained on the cell membrane despite the presence of α-factor secretion signal, whereas EGFP (enhance green fluorescent protein) was successfully secreted into culture medium (**Figure S6**).

**Figure 1:**
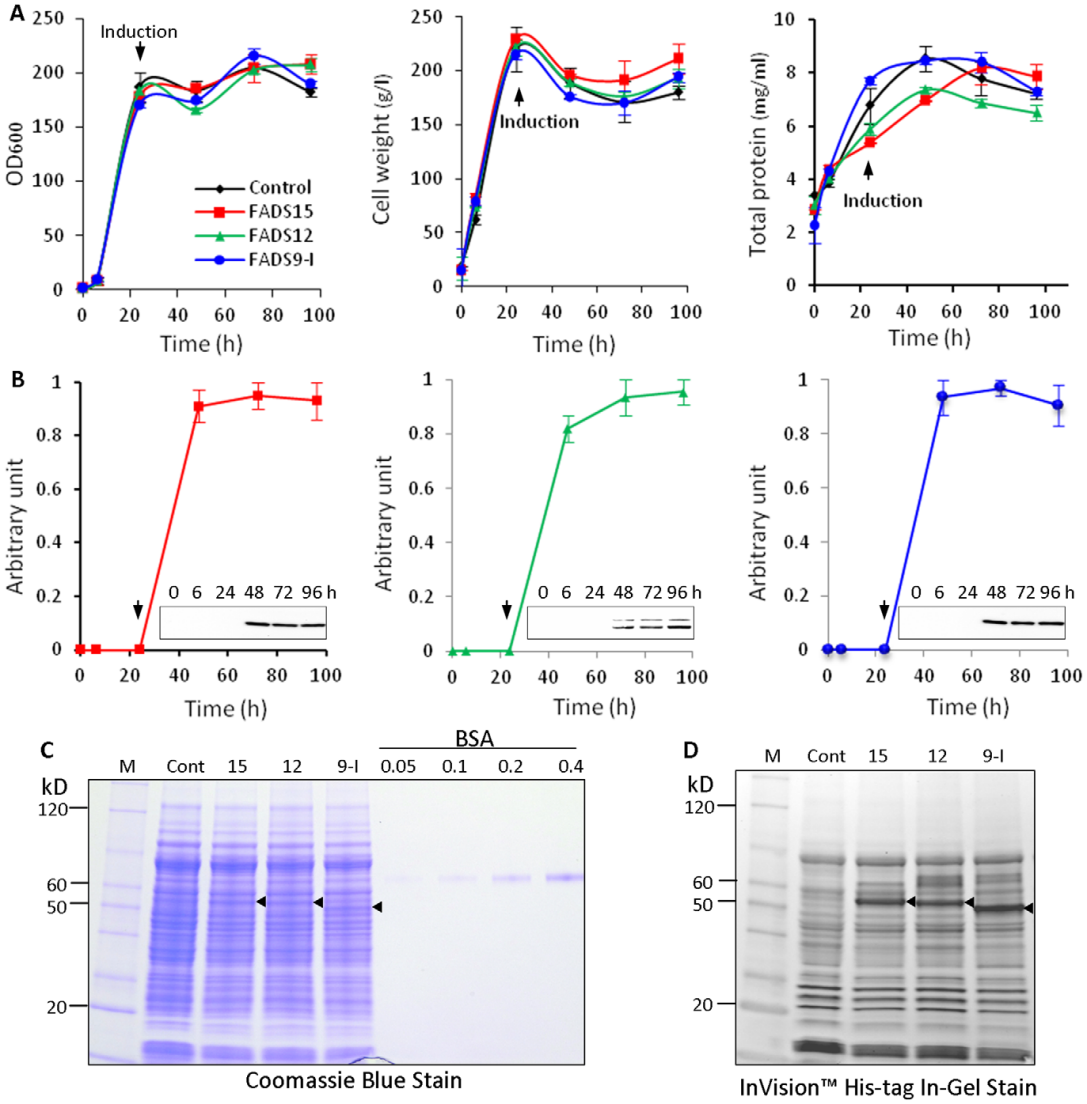
Expression of recombinant FADS. (**A**) Growth curve of the recombinant *P.pastoris* measured by cell density, wet weight and total protein concentration. (**B**) Kinetics of recombinant protein induction. Desaturase expression was determined by Western blotting using anti-His tag antibody. The normalized level of highest expression was set at one arbitrary unit. Three independent experiments were performed and bars represent standard deviations. (**C**) Quantification of the recombinant desaturase proteins by Coomassie blue staining after SDS-PAGE. Known concentrations of BSA were used as quantification standard. (**D**) InVision™ His-tag In-Gel Stain of recombinant FADS proteins. The arrow head indicates the addition of methanol for induction of recombinant protein expression. The triangles indicate the expressed recombinant proteins. M: protein marker, Cont: negative control which was PichiaPink™ harboring pPinkα-HC, 15: FADS15, 12: FADS12, 9-I: FADS9-I.

To determine potential toxicity of recombinant proteins, we first examined cell growth density, weight and total protein synthesis of the PichiaPink pPinkα-HC-FADS clones. The recombinant PichiaPink pPinkα-HC-FADS cells had growth characteristics similar to the control (**Figure 1A**). A time course experiment showed that desaturase expression was detectable after 24 hr induction with 0.5% methanol and remained high for at least 72 hr post-induction (**Figure 1B**). There were no significant differences in protein expression when cells were induced at different temperatures (16°C, 22°C, 28°C) or with a different concentration of methanol (0.5%, 1%). Therefore, we used an optimized procedure as described in the Materials and Methods for the expression of recombinant desaturase. Under this condition, expression levels of recombinant desaturase proteins reached approximately 130 mg/L of culture for FADS15, 110 mg/L for FADS12 and 350 mg/L for FADS9-I (**Figure 1C** **and** **Table 1**). FADS15 and FADS12 recombinant proteins overlapped with endogenous background proteins in Coomassie blue staining gels. The InVision™ His-tag In-Gel staining, however, showed clearly the expression of FADS15 and FADS12 over control (Figure 1D).

**Table 1:** Purification of *M. alpina* desaturases.

Step	FADS15 protein		FADS12 protein		FADS9-I protein	
	Conc. (mg/L)	Yield (%)	Conc. (mg/L)	Yield (%)	Conc. (mg/L)	Yield (%)
Cell lysate	130.0	100.0	110.0	100.0	350.0	100.0
Centrifugation (500–10,000 g)	112.0	86.2	82.0	72.7	254.0	72.6
Detergent extract	76.0	58.5	74.8	68.2	223.0	63.7
Ni-NTA 20 mM ID(5 mM ID)	4.6(5.5)	3.5(4.2)	2.5(4.0)	2.3(3.6)	37.5(92.4)	10.7(26.4)

ID: imidazole.

### Solubilizaton and Purification of Recombinant Desaturases

**Figure 2:**
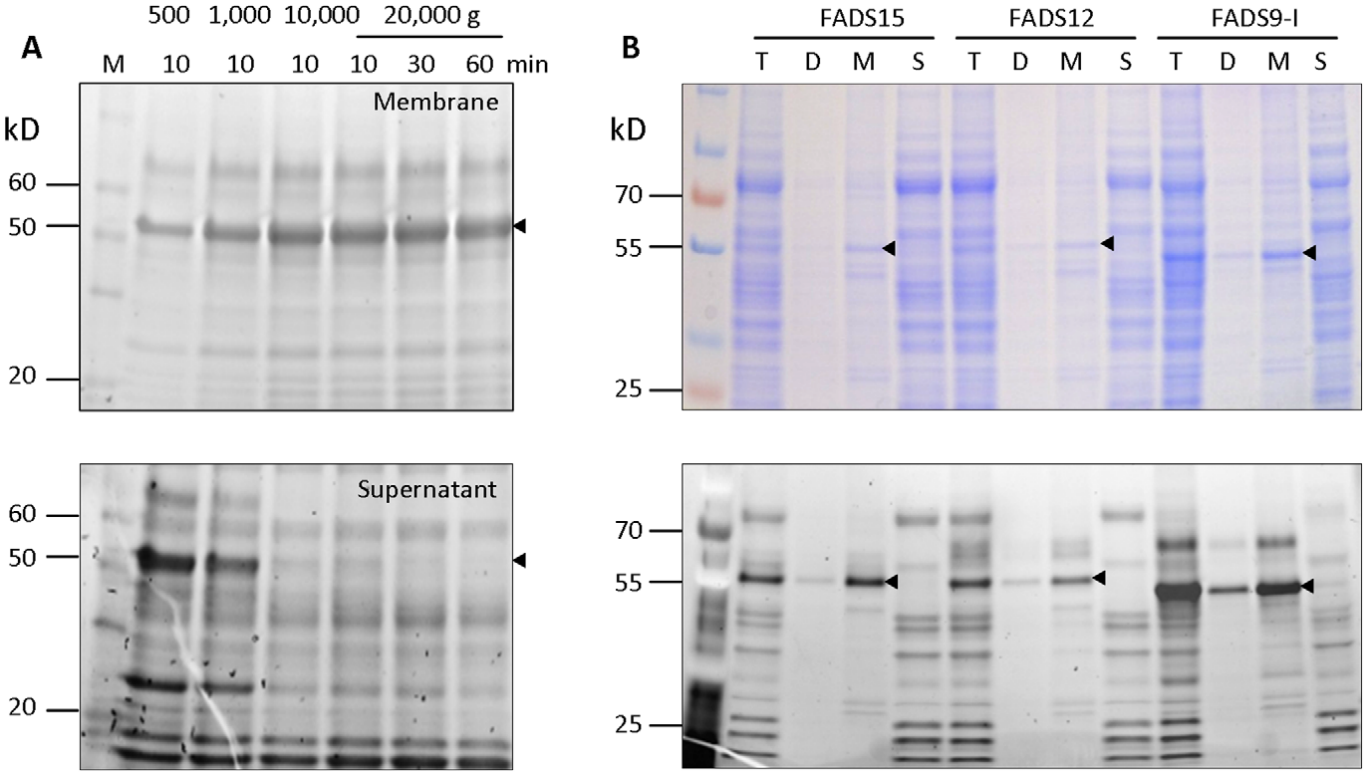
Fractionation of recombinant desaturases. (**A**) InVision™ His-tag In-Gel Staining after SDS-PAGE analysis of FADS9-I membrane (top panel) and supernatant fraction (bottom panel), using different speeds of centrifugation. The triangles indicate the recombinant FADS9-I. (**B**) Coomassie blue Staining and InVision™ His-tag In-Gel Staining of recombinant desaturases after fractionation. T: total protein after grinded by glass beads, D: debris after centrifugation at 500 g for 10 min, M: membrane fraction after centrifugation at 10,000 g for 10 min, S: supernatant after the centrifugation. The triangles indicate the recombinant desaturase proteins.

In order to solubilize and purify the recombinant desaturases from cell membrane for *in vitro* enzymatic activity, we first tested conditions to enrich the cell membrane containing recombinant FADS15, FADS12 and FADS9-I. Different centrifugation speeds and times were examined for the separation of the membrane fractions containing target proteins. Efficient recovery of each recombinant desaturase produced in *P. pastoris* was achieved by centrifuging the cell homogenates at 500 g for 10 min to remove cell debris, then at 10, 000 g for 10 min to collect membranes (**Figure 2**).

**Figure 3:**
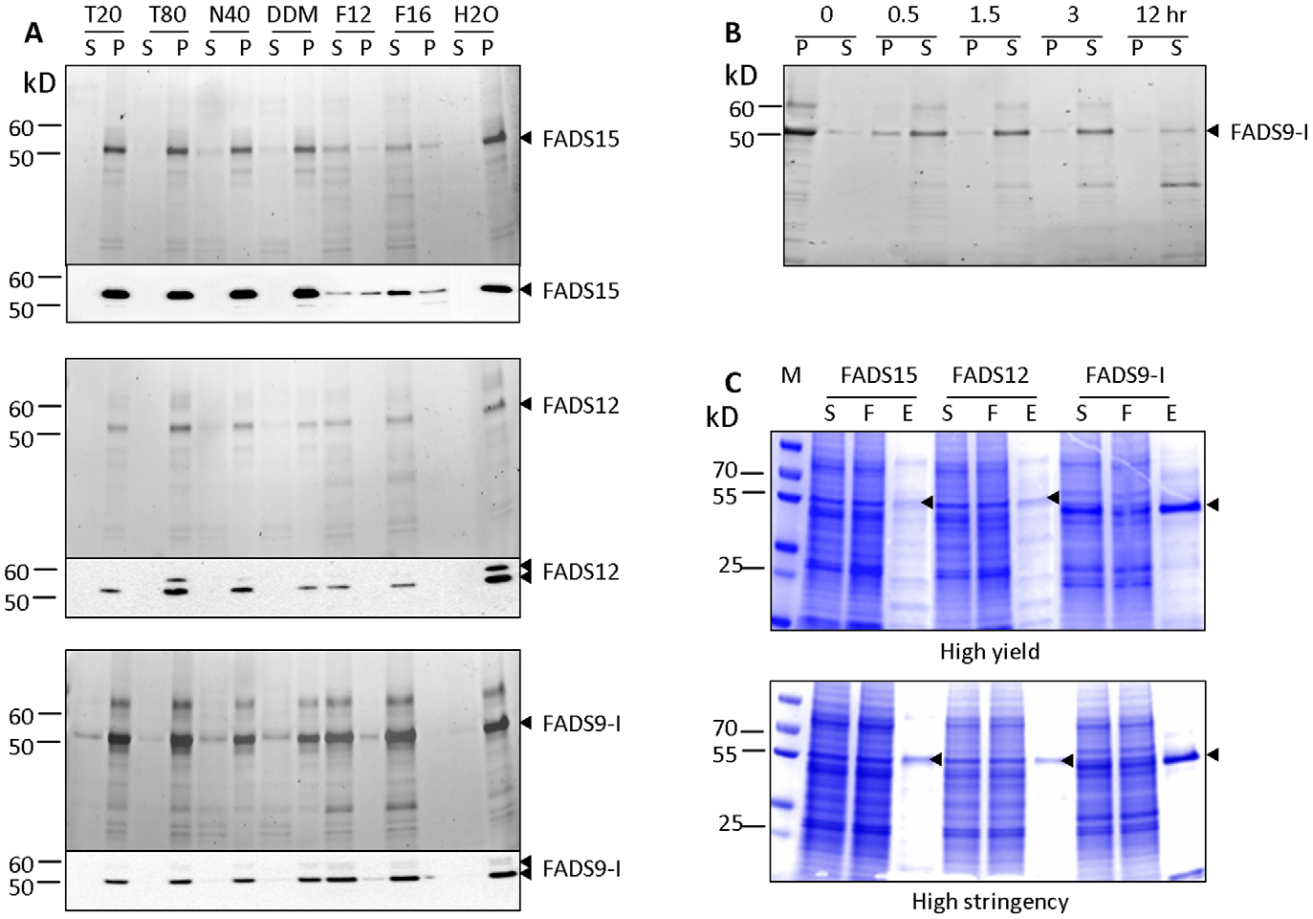
Solubilization of recombinant desaturases. (**A**) Membrane fractions were suspended in 1% concentrations of various detergents and incubated at 4°C for 2 hr. Proteins were visualized by InVision™ His-tag In-Gel Staining (upper panel) and Western blot (lower panel). T20: Tween-20, T80: Tween-80, N40: NP-40, DDΜ: *n-*Dodecyl*-β-*D-maltoside, F12: Fos-Choline 12, F16: Fos-Choline 16,S: supernatant, P: pellet. (**B**) Membrane fractions of recombinant FADS9-I were suspended in 1% Fos-Choline 16 and incubated at 4°C for various time (0, 0.5, 1.5, 3, 12 hr). Aliquots were analyzed by InVision™ His-tag In-Gel Staining. S: supernatant, P: pellet. (**C**) One-step purification using His Mag Sepharose Ni beads under the high yield (upper panel) and high stringency conditions (lower panel). Proteins were analyzed by SDS-PAGE and Coomassie blue staining. M: protein marker, S: supernatant, F: flow through, E: eluate.

Solubilization of membrane proteins requires the presence of detergents. Therefore, we tested the conditions for solubilization of the recombinant FADS15, FADS12 and FADS9-I from enriched cell membrane fractions using a panel of detergents: Tween-20, Tween-80, NP-40, n-Dodecyl-β-D-maltoside (DDM), Fos-Choline 12 or Fos-Choline 16. After treatment with 1% (w/v) of Fos-Choline 12 or Fos-Choline 16, FADS9-I and FADS12 were totally solubilized, and approximately 50% and 80% of FADS15 was solubilized with Fos-Choline 12 and Fos-Choline 16, respectively (**Figure 3A**). Tween-20, Tween-80, NP-40 and DDM had little effect on extracting these desaturase enzymes from the membrane. In addition, we noticed that FADS9-I protein degradation occurred during protein solubilization. This phenomenon was visible for proteins solubilized by both Fos-Choline 12 and 16. Thus, we investigated detergent incubation time during solubilization to optimize for the least protein degradation. Our results showed that the solubilization of FADS9-I protein reached its maximum level after incubation with detergent for 1.5 hr. Degradation of desaturase protein increased after more than 3 hr of incubation (**Figure 3B**). To maximize the ratio of intact vs. degraded proteins, we used 1.5 hr as our standard detergent incubation time for protein solubilization. We also compared the effect of detergent concentrations on protein solubilization efficiency and found that 0.5%, 1% or 2% of Fos-Choline 16 had similar effects. Taken together, our results indicate that all three recombinant desaturase enzymes can be solubilized efficiently from the cell membrane with 0.5% Fos-Choline 16 for 1.5 hr at 4°C.

Solubilized FADS15, FADS12 and FADS9-I were affinity-purified on His Mag Sepharose Ni beads (GE healthcare) with aims of high purity or high yield. High purity (>95%) was achieved after one step purification using the His Mag Sepharose Ni beads with high stringency wash before elution (**Figure 3C,** **S7**). High yield (2-fold higher than that in the high purity process) was achieved with low stringency wash. Yield and quantity of each desaturase enzyme are summarized in **Table 1**. Our estimated yields of desaturases with purity >95% are approximately 3.5% (Ca. 4.6 mg/L of culture) for FADS15, 2.3% (Ca. 2.5 mg/L of culture) for FADS12 and 10.7% (Ca. 37.5 mg/L of culture) for FADS9-I.

### In vivo and in vitro Analyses of Recombinant Desaturase Activities

To determine the functional activity of the recombinant *M. alpina* desaturase *in vivo*, PichiaPink cells were cultured and induced to express desaturases. Fatty acid methyl esters (FAME) analysis of cell pellets showed that expression of recombinant desaturases in PichiaPink cells altered their fatty acid contents compared to the control (typical samples data are shown in Table S2). **Table 2** shows the percentage increase of C16∶1^ Δ9^, C18∶1^ Δ9^, C18∶2^ Δ9,12^ and C18∶2^ Δ9,12,15^ compared to the negative control. The C16∶1^ Δ9^ and C18∶1^ Δ9^ were increased 40% and 20%, respectively, in PichiaPink cells expressing FADS9-I, suggesting that FAD9-I can insert the first double bond into both C16∶0 and C18∶0 with a preference for C16∶0 as substrate. The C18∶2^ Δ9,12^ content was 27% higher in cells expressing FADS12, suggesting that FADS12 can desaturate C18∶1^ Δ9^ at the c12-position to produce C18∶2^Δ9,12^. There was a 5% increase in C18∶3^Δ9,12,15^ in cells expressing FADS15, suggesting that FADS15 can desaturate C18∶2 ^Δ9,12^ at the c15-position to produce C18∶3^Δ9,12,15^. These results suggest that the recombinant desaturases, FADS9-I, FADS12 and FADS15, were active in *P. pastoris*.

**Table 2:** Calculated conversion rate of *M. alpina* FADS9-I, FADS12 and FADS15.

*In vivo*				*In vitro*			
FADS9-I		FADS12	FADS15	FADS9-I		FADS12	FADS15
C16∶1^Δ9^ (%^a^)	C18∶1^Δ9^ (%)	C18∶2^Δ9,12^ (%)	C18∶3^Δ9,12,15^ (%)	C16∶1^Δ9^ (%)	C18∶1^Δ9^ (%)	C18∶2^Δ9,12^ (%)	C18∶3^Δ9,12,15^ (%)
40±6	20±7	27±4	5±3	6±8	7±7	116±40	8±4
60±7^b^				13±8			

a Percent increase over control;

b Sum of two products.

*Saccharomyces cerevisiae* is an excellent experimental system to study fatty acid desaturation, as it provides a eukaryotic endoplasmic reticulum, cytochrome b5 and NADH and lacks polyunsaturated fatty acid [20]. We used yeast EGY49 cell homogenate for our *in vitro* assay of recombinant desaturase activity. Our results showed that purified recombinant FADS12 converted C18∶1^Δ9^ to C18∶2^ Δ9,12^ *in vitro*, and C18∶2^ Δ9,12^ level was increased 116% compared to the control (**Table 2**). Activities of purified FADS9-I and FADS15 were relatively low *in vitro*.

**Figure 4:**
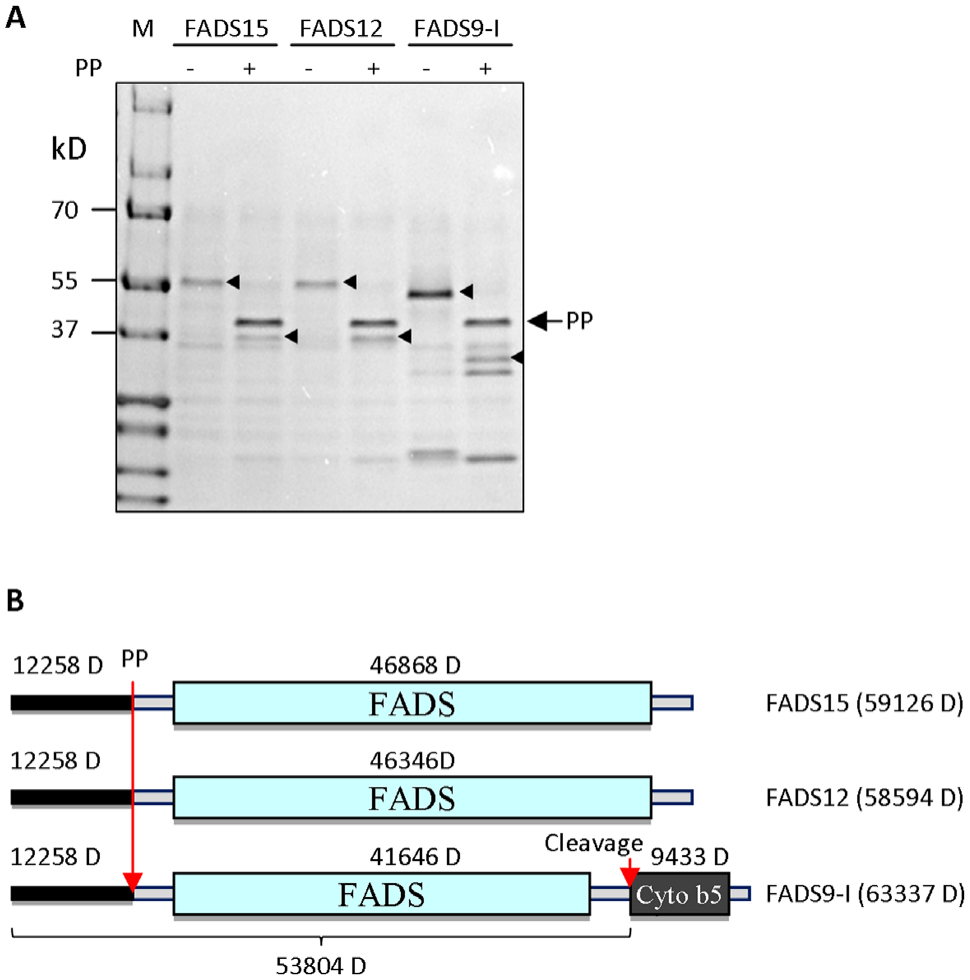
Precision protease (PP) digestion of recombinant desaturases. (**A**) Recombinant FADS proteins were digested with PP and visualized by Coomassie blue staining. Triangles indicate the recombinant desaturases before and after PP digestion. Arrow indicates Precision protease proteins. M: protein marker. (**B**) Diagram of recombinant desaturase structures with indicated molecular weight and cleavage sites. Long arrow indicates the Precision protease digestion site. Short arrow indicates the cleavage site before the Cyto b5 domain.

We noticed that the size of FADS9-I was smaller on SDS-PAGE than its predicted molecular weight. To determine whether a cleavage had occurred on the N-terminus which has a His-tag and Precision protease (PP) cleave site, the purified recombinant proteins were digested with PP (GE Healthcare) and analyzed by Coomassie staining of SDS-PAGE gel. The reduction in molecular weight supported that all three desaturases had the His-tag and Precision protease cleave site which were removed by PP digestion. Therefore, FADS9-I might have been cleaved on its C-terminus. Molecular weight of the FADS9-I protein was determined by mass spectrometry. Result suggests that the cytochrome b5 domain was cleaved off from FADS9-I during protein solubilization and purification (**Figure 4**).

## Discussion

No structural information is available to explain the regio-and substrate-selectivity of the membrane class of fatty acid desaturases. Our initial goal was to express high amounts of recombinant desaturase as secreted proteins in the methylotrophic yeast *Pichia pastoris*. The pre-pro α-factor from *Saccharomyces cerevisiae*, the most commonly used signal sequence for targeting protein secretion, was used in our recombinant desaturase expression. However, the expressed FADS proteins remained membrane-bound, whereas recombinant EGFP (enhance green fluorescent protein) proteins were efficiently secreted in our expression system. This membrane association of FADS proteins necessitates an effective solubilization procedure for protein purification.

There is no reference that we can find in the literature about detergents used for solubilization of integral membrane desaturases. Detergents, such as Tween-20, Tween-80, NP-40, and DDM have been commonly used for solubilization of membrane proteins, and their efficiency could be explained by their polar head group structure (**Figure S8**). Our results showed that none of them had any effect on solubilization of our recombinant FADS. Instead, we found that FADS9-I and FADS12, and FADS15 were efficiently solubilized in detergent Fos-Choline 12 and 16. It is possible that the structural similarity of Fos-Choline 12 and 16 to fatty acids may contribute to their ability to solubilize membrane fatty acid desaturase. The Fos-Choline detergents have also been successfully used in other membrane protein studies [21].

*In vivo* analysis showed high desaturase activity of FADS9-I. In contrast, the purified FADS9-I had relatively low desaturase activity. Some fatty acid desaturases possess a cytochrome b5 domain (**Figure S1**) used for electron transfer, whereas others do not have such domain and rely on external source of cytochrome b5. The molecular weight of purified FADS9-I was smaller than the predicted, and mass spectrometry data indicated a spontaneous removal of the cytochrome b5 domain (**Figure 4**). Therefore, the loss of internal cytochrome b5 may explain the low FADS9-I activity *in vitro*. FADS12, on the other hand, uses external source of cytochrome b5, and thus, retains high activity.

Sequence alignment showed that the FADS15 from *M. alpina* ATCC#32222 has eight amino acids differences (2.1%) compared to *M. alpina* 1s-4, which may affect substrate specificity. Typically, ω3 desaturases convert C18∶2^Δ9,12^ to C18∶3^Δ9,12,15^. Expression of the FADS15 from *M. alpina* ATCC#32222 resulted in a moderate increase in C18∶3^Δ9,12,15^. Adding various amounts of C18∶2^Δ9,12^ fatty acid into Pichia culture or EGY49 yeast culture did not significantly increase the level of C18∶2^Δ9,12^ and thus the production of C18∶3^Δ9,12,15^. Meanwhile, the amount of C18∶0 and C18∶1^Δ9^ was decreased, suggesting that the FADS15 may use C18∶0 and C18∶1^Δ9^ as substrates as well.

Cloning, expression, purification and functional characterization of FADS9-I, FADS12 and FADS15 from *M. alpina* ATCC#32222 represent a critical step towards the structural elucidation of membrane fatty acid desaturases. *Mortierella alpina* is an oleaginous fungus which can produce lipids accounting for up to 50% of its dry weight in the form of triacylglycerols. It is used commercially for the production of arachidonic acid. Understanding the regio-and substrate-selectivity of membrane class fatty acid desaturases may also be useful for the genetic engineering of strains producing higher levels and different constituents of dietary fat.

## Supporting Information

Figure S1 **Fatty acid desaturase identified in** ***M. alpina*** **ATCC#32222.** (**A**) Fatty acid synthesis pathway. Enzymes involved in this pathway are indicated in red. Desaturase studied in this paper are highlighted in yellow. ACC: acetyl-CoA carboxylase, ELOVL: fatty acid elongase, FASN: fatty acid synthase, ACSL: acyl-CoA synthetase, FADS9: fatty acid delta 9 desaturase, FADS12: fatty acid delta 12 desaturase, FADS15: fatty acid delta 15 desaturase, FADS6: fatty acid delta 6 desaturase, FADS5: fatty acid delta 5 desaturase. (**B**) Diagram of desaturase structures. FADS: fatty acid desaturase domain. Cyto b5: cytochrome b5 domain.
(TIF)

Figure S2 **Diagram of the cloning strategy for desaturase expression vectors.** FADS coding sequences were PCR amplified using primers listed in **Table S1**. PCR fragment were digested with indicated restriction enzymes, column purified and inserted into the pET-19b(PP) vector linearized with corresponding restriction enzymes. The FADS coding sequence plus His tag and Precision protease recognition sequence were PCR amplified and inserted into the pPinkalpha-HC vector. TRP2: TRP2 gene, AmpR: ampicillin resistance gene, pUC ori: oriental promoter of pUC, PAOX1∶5′AOX1 promoter region, α-factor: α-mating factor secretion signal, CYC1 TT: CCY1 transcription termination region, PADE2 HC: high-copy ADE2 promoter region, ADE2: ADE2 open reading frame.
(TIF)

Figure S3 **Alignment of FADS9-I coding sequences from different strains of** ***M. alpina*** **.** Differences in nucleotide or amino acid are highlighted.
(PDF)

Figure S4 **Alignment of FADS12 coding sequences from different strains of** ***M. alpina*** **.** Differences in nucleotide or amino acid are highlighted.
(PDF)

Figure S5 **Alignment of FADS15 coding sequences from different strains of** ***M. alpina*** **.** Differences in nucleotide or amino acid are highlighted.
(PDF)

Figure S6 **Membrane association of recombinant desaturase proteins.** PichiaPink cells were cultured for 24 hr, and induced with 0.5% methanol for 48 hr. Cell pellet and culture medium were analyzed by Western blot using anti-His tag antibody. Recombinant desaturase proteins were present exclusively in the cells fraction whereas EGFP protein was secreted into the culture medium. M: protein marker, Cont: PichiaPink cell harboring pPinkalpha-HC vector, EGFP: PichiaPink cell harboring pPinkalpha-HC-EGFP vector. C: cells, M: medium.
(TIF)

Figure S7 **Quantification of the recombinant desaturase proteins after one-step purification.** Known concentrations of BSA were used as quantification standard. Proteins were analyzed by SDS-PAGE and Coomassie blue staining. Protein purity was calculated by dividing the amount of a given desaturase protein quantified on gel by the amount of loaded protein quantified by the Pierce BCA protein assay. M: protein marker, S: supernatant, F: flow through, E: eluate.
(TIF)

Figure S8 **Structures of detergents used in the experiments.**
(TIF)

Table S1 **Primers for PCR reaction.**
(DOC)

Table S2 **FAME analysis of recombinant desaturase-expressing PichiaPink cells.**
(DOC)

## References

[1] Shanklin J, Somerville C (1991) Stearoyl-acyl-carrier-protein desaturase from higher plants is structurally unrelated to the animal and fungal homologs. Proc Natl Acad Sci U S A 88: 2510–2514. 2006187 10.1073/pnas.88.6.2510 PMC51262

[2] Shanklin J, Cahoon EB (1998) Desaturation and Related Modifications of Fatty Acids1. Annu Rev Plant Physiol Plant Mol Biol 49: 611–641. 15012248 10.1146/annurev.arplant.49.1.611

[3] Bloch K (1969) Enzymatic synthesis of monounsaturated fatty acids. Accounts of Chemical Research 2: 193–202.

[4] Berquin IM, Edwards IJ, Kridel SJ, Chen YQ (2011) Polyunsaturated fatty acid metabolism in prostate cancer. Cancer Metastasis Rev 30: 295–309. 22015690 10.1007/s10555-011-9299-7 PMC3865857

[5] Chen YQ, Edwards IJ, Kridel SJ, Thornburg T, Berquin IM (2007) Dietary fat-gene interactions in cancer. Cancer Metastasis Rev 26: 535–551. 17849170 10.1007/s10555-007-9075-x

[6] Hibbett DS, Binder M, Bischoff JF, Blackwell M, Cannon PF, et al (2007) A higher-level phylogenetic classification of the Fungi. Mycol Res 111: 509–547. 17572334 10.1016/j.mycres.2007.03.004

[7] Wang L, Chen W, Feng Y, Ren Y, Gu Z, et al (2011) Genome Characterization of the Oleaginous Fungus Mortierella alpina. PLoS One 6: e28319. 22174787 10.1371/journal.pone.0028319 PMC3234268

[8] Michaelson LV, Lazarus CM, Griffiths G, Napier JA, Stobart AK (1998) Isolation of a Delta5-fatty acid desaturase gene from Mortierella alpina. J Biol Chem 273: 19055–19059. 9668087 10.1074/jbc.273.30.19055

[9] Knutzon DS, Thurmond JM, Huang YS, Chaudhary S, Bobik EG Jr, et al (1998) Identification of Delta5-desaturase from Mortierella alpina by heterologous expression in Bakers’ yeast and canola. J Biol Chem 273: 29360–29366. 9792636 10.1074/jbc.273.45.29360

[10] Sakuradani E, Shimizu S (2003) Gene cloning and functional analysis of a second delta 6-fatty acid desaturase from an arachidonic acid-producing Mortierella fungus. Biosci Biotechnol Biochem 67: 704–711. 12784608 10.1271/bbb.67.704

[11] Sakuradani E, Kobayashi M, Ashikari T, Shimizu S (1999) Identification of Delta12-fatty acid desaturase from arachidonic acid-producing mortierella fungus by heterologous expression in the yeast Saccharomyces cerevisiae and the fungus Aspergillus oryzae. Eur J Biochem 261: 812–820. 10215899 10.1046/j.1432-1327.1999.00333.x

[12] MacKenzie DA, Carter AT, Wongwathanarat P, Eagles J, Salt J, et al (2002) A third fatty acid delta9-desaturase from Mortierella alpina with a different substrate specificity to ole1p and ole2p. Microbiology 148: 1725–1735. 12055292 10.1099/00221287-148-6-1725

[13] Sakuradani E, Abe T, Iguchi K, Shimizu S (2005) A novel fungal omega3-desaturase with wide substrate specificity from arachidonic acid-producing Mortierella alpina 1S-4. Appl Microbiol Biotechnol 66: 648–654. 15538555 10.1007/s00253-004-1760-x

[14] Kim SH, Kim JB, Kim SY, Roh KH, Kim HU, et al (2011) Functional characterization of a delta 6-desaturase gene from the black seabream (Acanthopagrus schlegeli). Biotechnology letters 33: 1185–1193. 21318631 10.1007/s10529-011-0555-2

[15] Li M, Ou X, Yang X, Guo D, Qian X, et al (2011) Isolation of a novel C18-Delta9 polyunsaturated fatty acid specific elongase gene from DHA-producing Isochrysis galbana H29 and its use for the reconstitution of the alternative Delta8 pathway in Saccharomyces cerevisiae. Biotechnology letters 33: 1823–1830. 21538137 10.1007/s10529-011-0626-4

[16] Goren MA, Fox BG (2008) Wheat germ cell-free translation, purification, and assembly of a functional human stearoyl-CoA desaturase complex. Protein expression and purification 62: 171–178. 18765284 10.1016/j.pep.2008.08.002 PMC2586813

[17] Jonsson TJ, Johnson LC, Lowther WT (2009) Protein engineering of the quaternary sulfiredoxin.peroxiredoxin enzyme.substrate complex reveals the molecular basis for cysteine sulfinic acid phosphorylation. J Biol Chem 284: 33305–33310. 19812042 10.1074/jbc.M109.036400 PMC2785173

[18] Bligh EG, Dyer WJ (1959) A rapid method of total lipid extraction and purification. Can J Biochem Physiol 37: 911–917. 13671378 10.1139/o59-099

[19] Metcalfe LD, Schmitz AA, Pelka JR (1966) Rapid preparation of fatty acids esters from lipids for gas chromatographic analysis. Analytical Chemistry 38: 514–515.

[20] Reed DW, Schafer UA, Covello PS (2000) Characterization of the Brassica napus extraplastidial linoleate desaturase by expression in Saccharomyces cerevisiae. Plant Physiol 122: 715–720. 10712534 10.1104/pp.122.3.715 PMC58906

[21] Oxenoid K, Chou JJ (2005) The structure of phospholamban pentamer reveals a channel-like architecture in membranes. Proc Natl Acad Sci U S A 102: 10870–10875. 16043693 10.1073/pnas.0504920102 PMC1182456

