# Supplementary figures and images for: Expression and Purification of Integral Membrane Fatty Acid Desaturases
Source: PLoS One. 2013 Mar 8;8(3):e58139. doi: 10.1371/journal.pone.0058139 (PMC3592867; doi:10.1371/journal.pone.0058139)

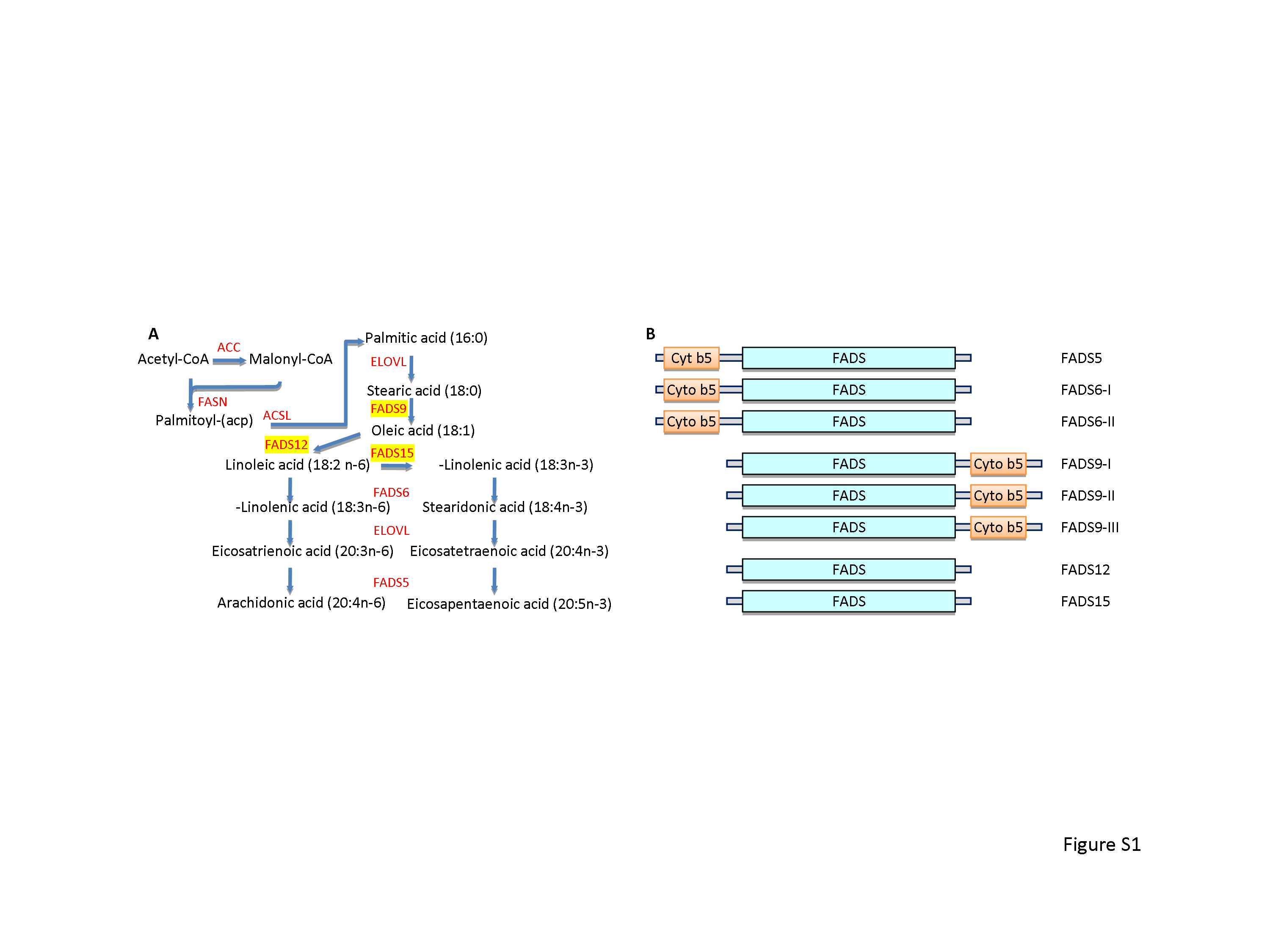

Supplement: Figure S1 — Fatty acid desaturase identified in M. alpina ATCC#32222. (A) Fatty acid synthesis pathway. Enzymes involved in this pathway are indicated in red. Desaturase studied in this paper are highlighted in yellow. ACC: acetyl-CoA carboxylase, ELOVL: fatty acid elongase, FASN: fatty acid synthase, ACSL: acyl-CoA synthetase, FADS9: fatty acid delta 9 desaturase, FADS12: fatty acid delta 12 desaturase, FADS15: fatty acid delta 15 desaturase, FADS6: fatty acid delta 6 desaturase, FADS5: fatty acid delta 5 desaturase. (B) Diagram of desaturase structures. FADS: fatty acid desaturase domain. Cyto b5: cytochrome b5 domain. (TIF) [file pone.0058139.s001.tif]

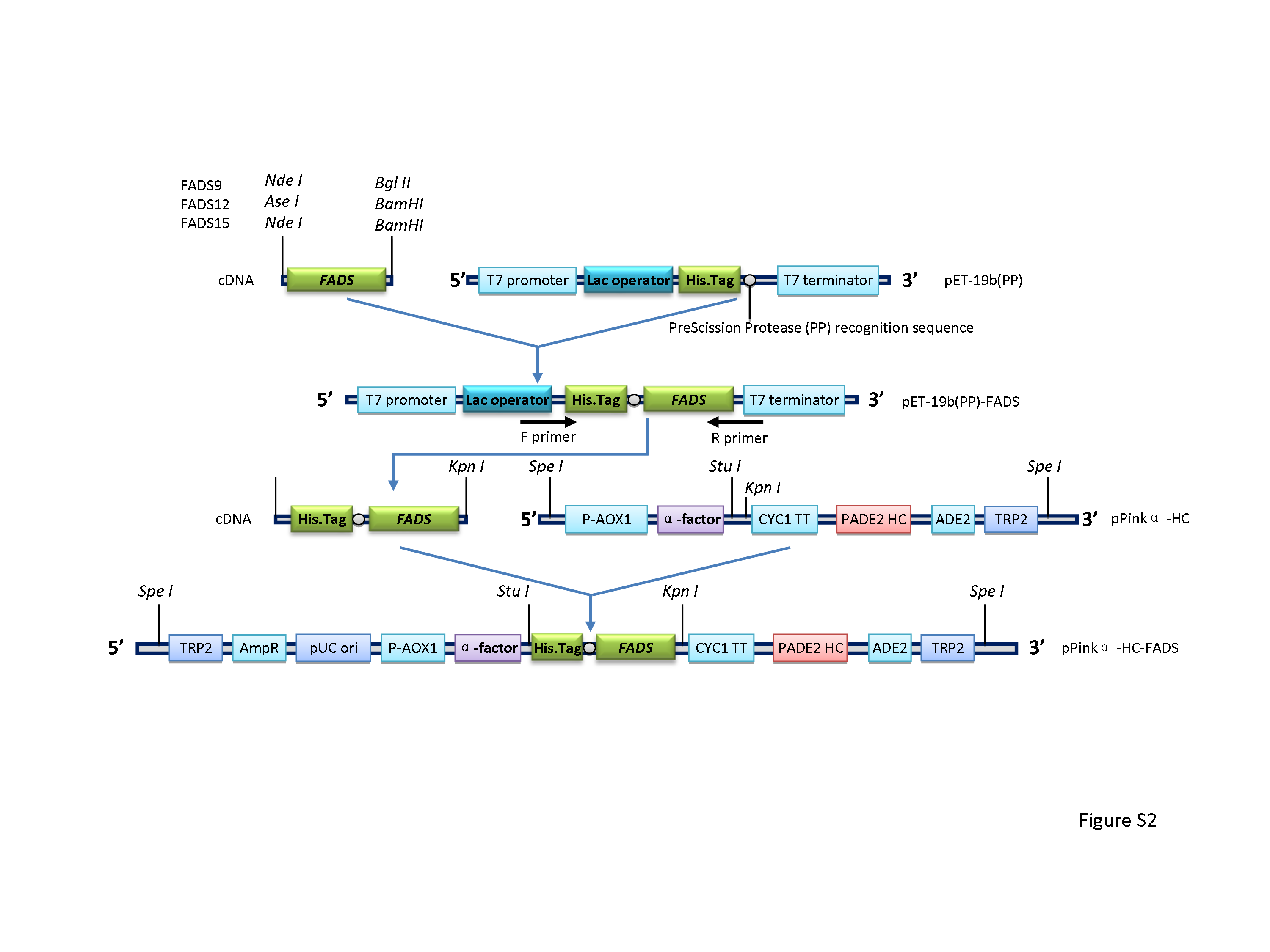

Supplement: Figure S2 — Diagram of the cloning strategy for desaturase expression vectors. FADS coding sequences were PCR amplified using primers listed in Table S1. PCR fragment were digested with indicated restriction enzymes, column purified and inserted into the pET-19b(PP) vector linearized with corresponding restriction enzymes. The FADS coding sequence plus His tag and Precision protease recognition sequence were PCR amplified and inserted into the pPinkalpha-HC vector. TRP2: TRP2 gene, AmpR: ampicillin resistance gene, pUC ori: oriental promoter of pUC, PAOX1∶5′AOX1 promoter region, α-factor: α-mating factor secretion signal, CYC1 TT: CCY1 transcription termination region, PADE2 HC: high-copy ADE2 promoter region, ADE2: ADE2 open reading frame. (TIF) [file pone.0058139.s002.tif]

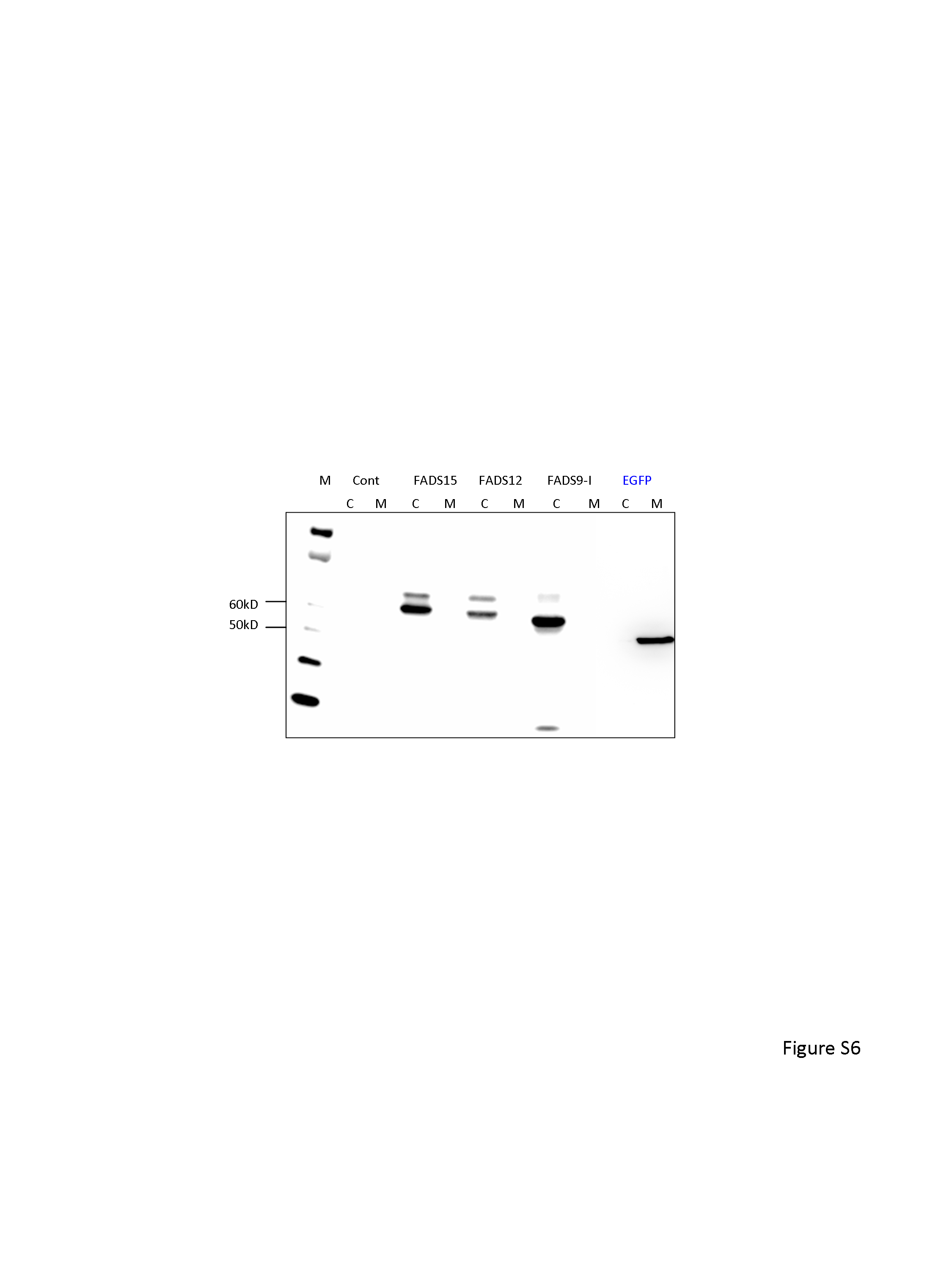

Supplement: Figure S6 — Membrane association of recombinant desaturase proteins. PichiaPink cells were cultured for 24 hr, and induced with 0.5% methanol for 48 hr. Cell pellet and culture medium were analyzed by Western blot using anti-His tag antibody. Recombinant desaturase proteins were present exclusively in the cells fraction whereas EGFP protein was secreted into the culture medium. M: protein marker, Cont: PichiaPink cell harboring pPinkalpha-HC vector, EGFP: PichiaPink cell harboring pPinkalpha-HC-EGFP vector. C: cells, M: medium. (TIF) [file pone.0058139.s006.tif]

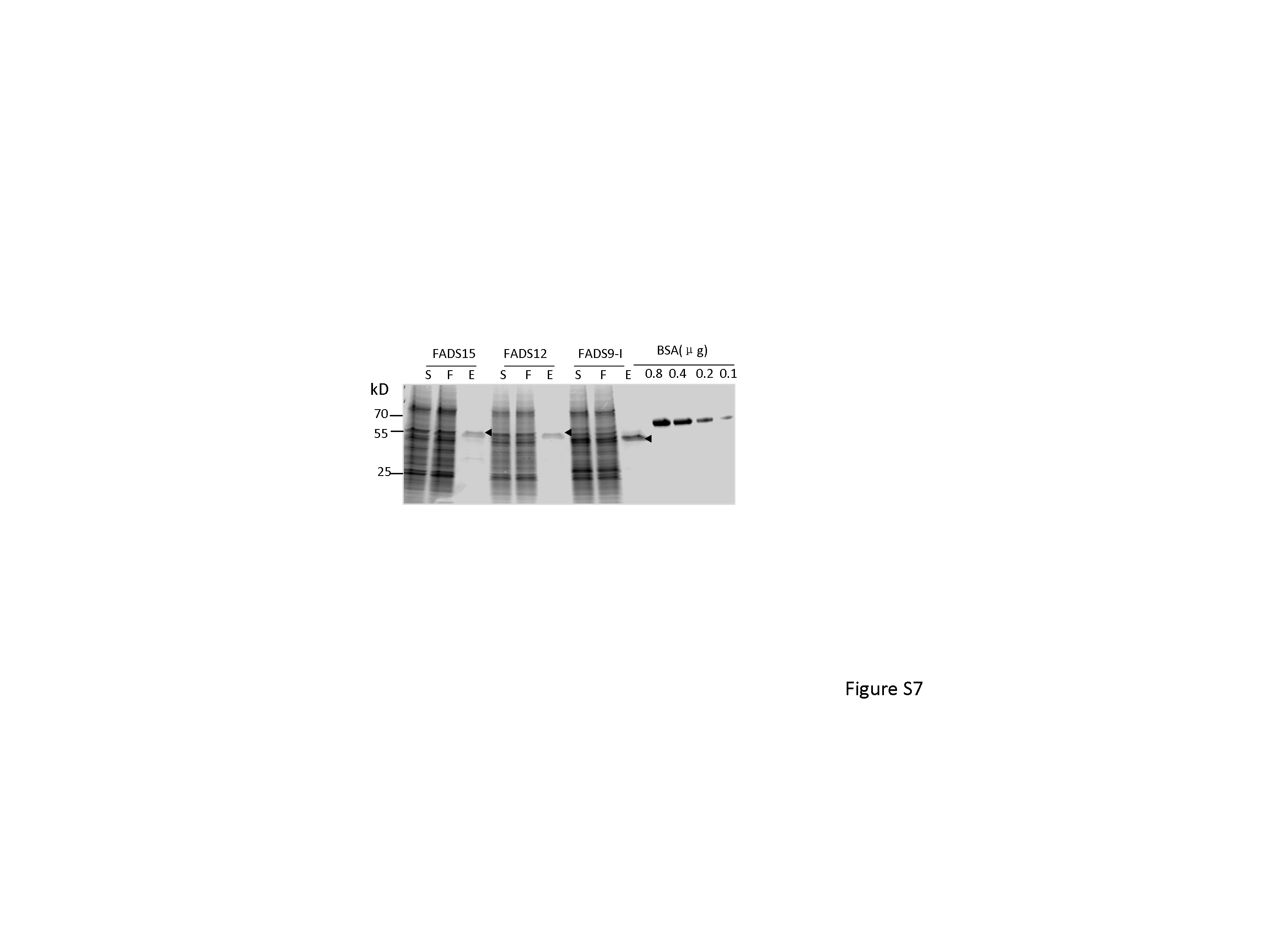

Supplement: Figure S7 — Quantification of the recombinant desaturase proteins after one-step purification. Known concentrations of BSA were used as quantification standard. Proteins were analyzed by SDS-PAGE and Coomassie blue staining. Protein purity was calculated by dividing the amount of a given desaturase protein quantified on gel by the amount of loaded protein quantified by the Pierce BCA protein assay. M: protein marker, S: supernatant, F: flow through, E: eluate. (TIF) [file pone.0058139.s007.tif]
