# Supplementary material for: Expression and Purification of Integral Membrane Fatty Acid Desaturases
Source: PLoS One. 2013 Mar 8;8(3):e58139. doi: 10.1371/journal.pone.0058139 (PMC3592867; doi:10.1371/journal.pone.0058139)
Supplement: Figure S3 — Alignment of FADS9-I coding sequences from different strains of M. alpina . Differences in nucleotide or amino acid are highlighted. (PDF) [file pone.0058139.s003.pdf]

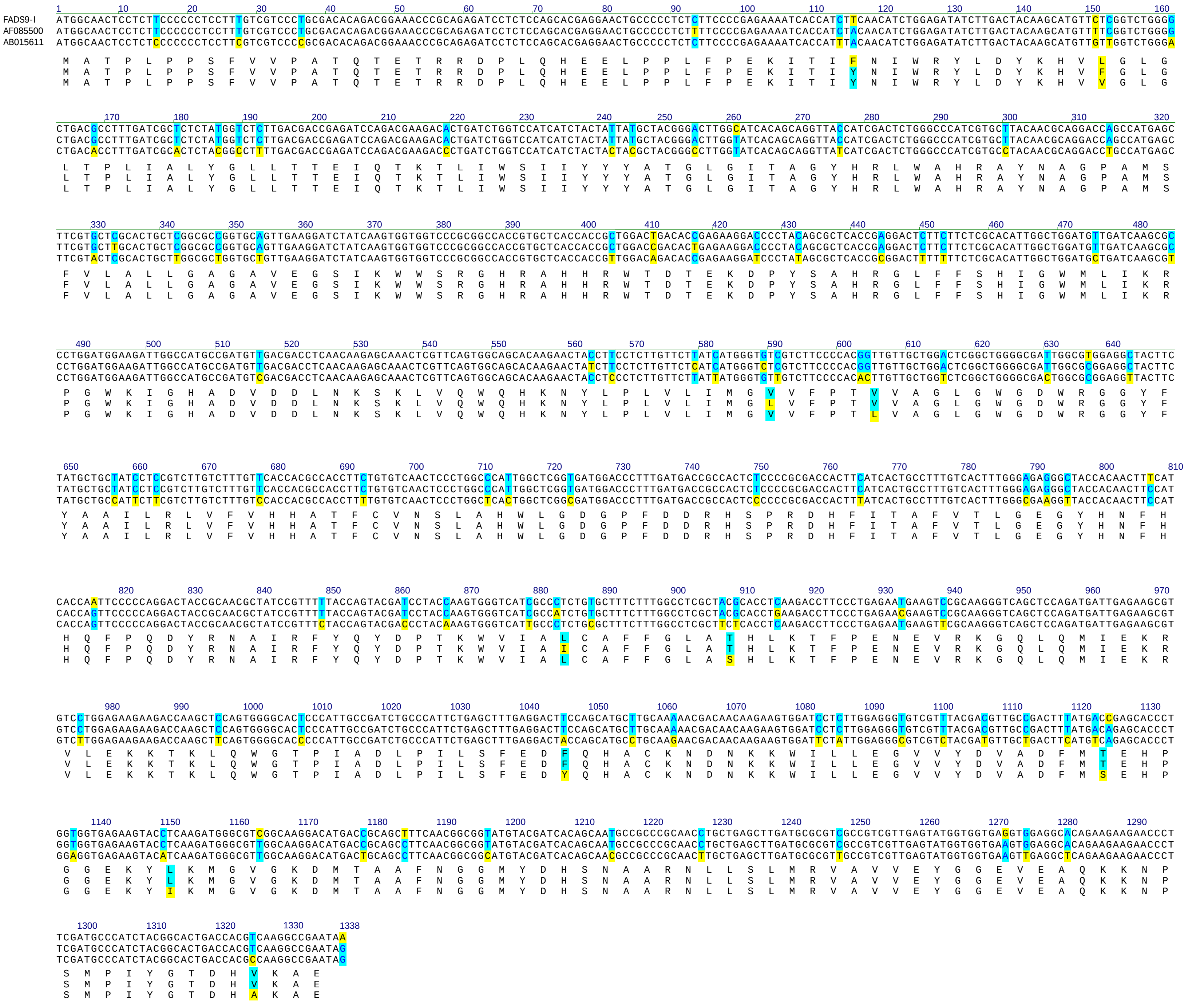

Figure S3
