# Supplementary material for: Expression and Purification of Integral Membrane Fatty Acid Desaturases
Source: PLoS One. 2013 Mar 8;8(3):e58139. doi: 10.1371/journal.pone.0058139 (PMC3592867; doi:10.1371/journal.pone.0058139)
Supplement: Table S1 — Primers for PCR reaction. (DOC) [file pone.0058139.s009.doc]

Table S1. Primers for PCR reaction

| Name | Sequence | Targeted vector |
| --- | --- | --- |
| FF1 | atat*CATATG*ATGGCCCCCCCTCACGTTGTCGACGAGCA ***(Nde I)*** | pET19b-FADS15 |
| FF2 | atat*ATTAAT*ATGGCACCTCCCAACACTATTGATGCCGG ***(Ase I)*** | pET19b-FADS12 |
| FF3 | atat*CATATG*ATGGCAACTCCTCTTCCCCCCTCCTTTGT ***(Nde I)*** | pET19b-FADS9-I |
| FR1 | atat*GGATCC*TAATGCTTGTAGAACACTACGTC ***(BamH I)*** | pET19b-FADS15 |
| FR2 | atat*GGATCC*TTACTTCTTGAAAAAGACCACGTC ***(BamH I)*** | pET19b-FADS12 |
| FR3 | atat*AGATCT*TTATTCGGCCTTGACGTGGTCAGT ***(Bgl II)*** | pET19b-FADS9-I |
| SF1 | atatatTGCGCACATCATCATCATCATCATCAT ***(Fsp I)*** | pPink-FADS |
| SR1 | atat*GAATTC*AT*ATTTAAAT*TAATGCTTGTAGAACACTACGTC ***(EcoR I, Swa I)*** | pPink-FADS15 |
| SR2 | atatat*GGTACC*TTACTTCTTGAAAAAGACCACGTC ***(Kpn I)*** | pPink-FADS12 |
| SR3 | atatat*GGTACC*TTATTCGGCCTTGACGTGGTCAGT ***(Kpn I)*** | pPink-FADS9-I |
