# Supplementary material for: Expression and Purification of Integral Membrane Fatty Acid Desaturases
Source: PLoS One. 2013 Mar 8;8(3):e58139. doi: 10.1371/journal.pone.0058139 (PMC3592867; doi:10.1371/journal.pone.0058139)
Supplement: Table S2 — FAME analysis of recombinant desaturase-expressing PichiaPink cells. (DOC) [file pone.0058139.s010.doc]

Table S2. FAME analysis of recombinant desaturase-expressing PichiaPink cells

| PichiaPink cells | 14:0* | 14:1 | 16:0 | 16:1 | 18:0 | total 18:1 | 18:1(oleate) | 18:1(vaccinate) | 18:2 | total 18:3 | 18:3 n-3 | 18:3 n-6 |
| --- | --- | --- | --- | --- | --- | --- | --- | --- | --- | --- | --- | --- |
| Control | 0.080 | 0.000 | 3.086 | 1.018 | 1.117 | 15.203 | 14.955 | 0.248 | 7.724 | 0.711 | 0.678 | 0.033 |
| FAD15 | 0.113 | 0.000 | 3.678 | 1.229 | 1.045 | 16.085 | 15.991 | 0.094 | 9.213 | 0.844 | **0.798** | 0.046 |
| FAD12 | 0.097 | 0.000 | 3.754 | 1.246 | 1.310 | 14.909 | 14.670 | 0.239 | **10.676** | 0.799 | 0.752 | 0.047 |
| FAD9-I | 0.134 | 0.000 | 3.964 | **1.457** | 1.178 | **19.858** | 19.438 | 0.420 | 8.144 | 0.778 | 0.731 | 0.047 |

* μg/mg cell weight
