# Supplementary material for: Expression and Purification of Integral Membrane Fatty Acid Desaturases
Source: PLoS One. 2013 Mar 8;8(3):e58139. doi: 10.1371/journal.pone.0058139 (PMC3592867; doi:10.1371/journal.pone.0058139)
Supplement: Figure S5 — Alignment of FADS15 coding sequences from different strains of M. alpina . Differences in nucleotide or amino acid are highlighted. (PDF) [file pone.0058139.s005.pdf]

```
          1        10        20        30        40        50        60        70        80        90        100       110       120       130       140       150       160
FADS15    ATGGCCCCCCCTCACGTTGTCGACGAGCAAGTACGACGCAGAATCGTCGTCGAGGACGAGATCCAGTCCAAGAAGCAGTTTGAGCGCAACTATGTGCCTATGGACTTTACAATCAAGGAGATTCGAGATGCGATCCCAGCCCACCTCTTCATCCGTGATACC
AB182163  ATGGCCCCCCCTCACGTTGTCGACGAACAAGTTCGACGCAGGATCGTCGTTGAGGACGAGATCAAGTCTAAGAAGCAATTTGAGCGCAACTATGTGCCCATGGACTTTACGATTAAGGAGATTCGAGATGCGATCCCTGCCCACCTCTTCATCCGTGATACC
DD291371  ATGGCCCCCCCTCACGTTGTCGACGAACAAGTTCGACGCAGGATCGTCGTTGAGGACGAGATCAAGTCTAAGAAGCAATTTGAGCGCAACTATGTGCCCATGGACTTTACGATTAAGGAGATTCGAGATGCGATCCCTGCCCACCTCTTCATCCGTGATACC
          M A P P H V V D E Q V R R R I V V E D E I Q S K K Q F E R N Y V P M D F T I K E I R D A I P A H L F I R D T
          M A P P H V V D E Q V R R R I V V E D E I K S K K Q F E R N Y V P M D F T I K E I R D A I P A H L F I R D T
          M A P P H V V D E Q V R R R I V V E D E I K S K K Q F E R N Y V P M D F T I K E I R D A I P A H L F I R D T

          170 180 190 200 210 220 230 240 250 260 270 280 290 300 310 320
          ACAAAGTCGATCCTGCATGTCGTCAAGGATCTGGTCACTATCGCCATCGTTTTTTACTGTGCAACCTTCATCGAGACTCTGCCCTCGCTCGCTCTGAGAGTTCCTGCCTGGATCACCTACTGGATCATCCAAGGAACTGTCATGGTCGGCCCCTGGATTCTG
          ACAAAGTCGATCCTGCATGTCGTCAAGGATCTGGTCACCATCGCCATCGTCTTTTACTGTGCAACCTTCATTGAGACTCTGCCCTCGCTCGCTCTGCGAGTTCCTGCCTGGATCACCTACTGGATCATCCAAGGAACTGTCATGGTCGGCCCCTGGATCTTG
          ACAAAGTCGATCCTGCATGTCGTCAAGGATCTGGTCACCATCGCCATCGTCTTTTACTGTGCAACCTTCATTGAGACTCTGCCCTCGCTCGCTCTGCGAGTTCCTGCCTGGATCACCTACTGGATCATCCAAGGAACTGTCATGGTCGGCCCCTGGATCTTG
          T K S I L H V V K D L V T I A I V F Y C A T F I E T L P S L A L R V P A W I T Y W I I Q G T V M V G P W I L
          T K S I L H V V K D L V T I A I V F Y C A T F I E T L P S L A L R V P A W I T Y W I I Q G T V M V G P W I L
          T K S I L H V V K D L V T I A I V F Y C A T F I E T L P S L A L R V P A W I T Y W I I Q G T V M V G P W I L

          330 340 350 360 370 380 390 400 410 420 430 440 450 460 470 480
          GCCCAC---------------------------------------------------------------------------------------------GAGTGCGGCCATGGAGCGTTCTCGGACAGCAAGACGATCAACACCATCTTTGGATGGGTCCTT
          GCTCAT---------------------------------------------------------------------------------------------GAGTGCGGCCACGGAGCTTTCTCGGATAGCAAGACGATCAACACCATCTTTGGATGGGTCCTC
          GCTCATGGTAAGGAAACGAAAAATCCCATGTGTATTTCTGTACTACAGAAGGCGAAGTTTGTACCTGAAAAGATCAGCGTCGTCCCTGTGTCAACGACAGAGTGCGGCCACGGAGCTTTCTCGGATAGCAAGACGATCAACACCATCTTTGGATGGGTCCTC
          A H - - - - - - - - - - - - - - - - - - - - - - - - - - - - - - - E C G H G A F S D S K T I N T I F G W V L
          A H - - - - - - - - - - - - - - - - - - - - - - - - - - - - - - - E C G H G A F S D S K T I N T I F G W V L
          A H G K E T K N P M C I S V L Q K A K F V P E K I S V V P V S T T E C G H G A F S D S K T I N T I F G W V L

          500 510 520 530 540 550 560 570 580 590 600 610 620 630 640
          CACTCTGCTCTTTTGGTGCCCTACCAGGCTTGGGCCATGTCGCATTCCAAGCACCACAAGGGCACTGGATCCATGAGCAAGGATGTCGTTTTCATCCCTGCCACTCGATCCTACAAGGGCCTTCCCCACTGGAGAAGCCTGCCGCGGAAGAGGAGGTTTTG
          CACTCTGCTCTTTTGGTGCCCTACCAGGCCTGGGCTATGTCACACTCCAAGCATCACAAGGGTACTGGATCGATGACCAAAGATGTCGTTTTCATCCCTGCCACTCGTTCCTACAAGGGCCTCCCAGCACTGGAGAAGCCTGCCGTCGAAGAGGAGGTTTCG
          CACTCTGCTCTTTTGGTGCCCTACCAGGCCTGGGCTATGTCACACTCCAAGCATCACAAGGGTACTGGATCGATGACCAAAGATGTCGTTTTCATCCCTGCCACTCGTTCCTACAAGGGCCTCCCAGCACTGGAGAAGCCTGCCGTCGAAGAGGAGGTTTCG
          H S A L L V P Y Q A W A M S H S K H H K G T G S M S K D V V F I P A T R S Y K G L P P L E K P A A E E E V L
          H S A L L V P Y Q A W A M S H S K H H K G T G S M T K D V V F I P A T R S Y K G L P A L E K P A V E E E V S
          H S A L L V P Y Q A W A M S H S K H H K G T G S M T K D V V F I P A T R S Y K G L P A L E K P A V E E E V S

          650 660 670 680 690 700 710 720 730 740 750 760 770 780 790 800 810
          GAGCAGGAGCATCACCACCATGAAGAGTCCATCTTTGCTGAGACTCCCATCTACACTCTCGGAGCGCTTTTTTTCGTCCTGACCTTGGGATGGCCCTTGTACTTGATCATGAACTTTTCTGGACACGAAGCCCCTCACTGGGTCAACCACTTCCAGACGGTC
          GAGCAGGAACACCACCACCACGAGGAGTCCATCTTTGCCGAAACTCCCATCTACACGCTCGGAGCGCTTTTGTTCGTCTTGACCTTCGGATGGCCCTTGTACTTGATCGTCAACTTTTCAGGACACGAGGCCCCTCACTGGGTCAACCATTTCCAGACTGTC
          GAGCAGGAACACCACCACCACGAGGAGTCCATCTTTGCCGAAACTCCCATCTACACGCTCGGAGCGCTTTTGTTCGTCTTGACCTTCGGATGGCCCTTGTACTTGATCGTCAACTTTTCAGGACACGAGGCCCCTCACTGGGTCAACCATTTCCAGACTGTC
          E Q E H H H H E E S I F A E T P I Y T L G A L F F V L T L G W P L Y L I M N F S G H E A P H W V N H F Q T V
          E Q E H H H H E E S I F A E T P I Y T L G A L L F V L T F G W P L Y L I V N F S G H E A P H W V N H F Q T V
          E Q E H H H H E E S I F A E T P I Y T L G A L L F V L T F G W P L Y L I V N F S G H E A P H W V N H F Q T V

          820 830 840 850 860 870 880 890 900 910 920 930 940 950 960 970
          GCCCCTCTGTATGAGCCTCACCAGCGCAAGAACATTTTCTACTCCAACTGCGGCATTGTCGCTATGGGCTCGATCCTCACTTACCTCTCGATGGTCTTCTCGCCCTTGACTGTGTTCATGTACTATGGCATCCCCTACCTCGGAGTCAATGCTTGGATCGTC
          GCTCCTCTCTATGAGCCTCACCAGCGCAAGAACATCTTCTACTCCAACTGCGGCATTGTCGCCATGGGTTCGATCTTGACTTACCTTTCGATGGTCTTCTCGCCCTTGACTGTCTTCATGTACTATGGCATCCCTTACCTCGGAGTCAACGCCTGGATCGTC
          GCTCCTCTCTATGAGCCTCACCAGCGCAAGAACATCTTCTACTCCAACTGCGGCATTGTCGCCATGGGTTCGATCTTGACTTACCTTTCGATGGTCTTCTCGCCCTTGACTGTCTTCATGTACTATGGCATCCCTTACCTCGGAGTCAACGCCTGGATCGTC
          A P L Y E P H Q R K N I F Y S N C G I V A M G S I L T Y L S M V F S P L T V F M Y Y G I P Y L G V N A W I V
          A P L Y E P H Q R K N I F Y S N C G I V A M G S I L T Y L S M V F S P L T V F M Y Y G I P Y L G V N A W I V
          A P L Y E P H Q R K N I F Y S N C G I V A M G S I L T Y L S M V F S P L T V F M Y Y G I P Y L G V N A W I V

          980 990 1000 1010 1020 1030 1040 1050 1060 1070 1080 1090 1100 1110 1120 1130
          TGCATCACCTATCTCCAGCACACCGATCCCAAGGTGCCTCATTTCCGTGATAACGAGTGGAACTTCCAGCGCGGTGCTGCCTGCACTATCGACCGATCCTTCGGTACCATTGTCAACCACTTGCACCACCACATTGGTGACTCTCATCAATGTCATCATATG
          TGCATTACCTATCTCCAGCACACCGATCCCAAGGTGCCTCACTTCCGTGATAACGAGTGGAACTTCCAGCGCGGTGCTGCCTGCACTATCGACCGATCCTTCGGTACCATCGTGAACCACCTGCACCACCACATTGGCGACTCTCACCAGTGCCACCATATG
          TGCATTACCTATCTCCAGCACACCGATCCCAAGGTGCCTCACTTCCGTGATAACGAGTGGAACTTCCAGCGCGGTGCTGCCTGCACTATCGACCGATCCTTCGGTACCATCGTGAACCACCTGCACCACCACATTGGCGACTCTCACCAGTGCCACCATATG
          C I T Y L Q H T D P K V P H F R D N E W N F Q R G A A C T I D R S F G T I V N H L H H H I G D S H Q C H H M
          C I T Y L Q H T D P K V P H F R D N E W N F Q R G A A C T I D R S F G T I V N H L H H H I G D S H Q C H H M
          C I T Y L Q H T D P K V P H F R D N E W N F Q R G A A C T I D R S F G T I V N H L H H H I G D S H Q C H H M

          1140 1150 1160 1170 1180 1190 1200 1210 1220 1230 1240 1250 1260 1270 1280 1290
          TTCTCGCAGATGCCCTTCTACAACGCCGTTGAGGCTACAAAGCATCTCAAAGCCAAGCTTGGCAAGTACTACATATTTGACGACACTCCCATTGCCAAGGCCCTCTACCGCAATTGGAGAGAGTGCAAATTCGTGGAGGACGAGGGAGACGTAGTGTTCTAC
          TTCTCGCAGATGCCCTTCTACAATGCTGTGGAGGCTACAAAGTACTTGAAGGCCAAACTTGGCAAGTACTACATATTTGACGACACGCCCATTGCCAAAGCCCTCTACCGCAATTGGAGAGAGTGCAAATTCGTGGAGGACGAGGGAGATGTAGTGTTTTAC
          TTCTCGCAGATGCCCTTCTACAATGCTGTGGAGGCTACAAAGTACTTGAAGGCCAAACTTGGCAAGTACTACATATTTGACGACACGCCCATTGCCAAAGCCCTCTACCGCAATTGGAGAGAGTGCAAATTCGTGGAGGACGAGGGAGATGTAGTGTTTTAC
          F S Q M P F Y N A V E A T K H L K A K L G K Y Y I F D D T P I A K A L Y R N W R E C K F V E D E G D V V F Y
          F S Q M P F Y N A V E A T K Y L K A K L G K Y Y I F D D T P I A K A L Y R N W R E C K F V E D E G D V V F Y
          F S Q M P F Y N A V E A T K Y L K A K L G K Y Y I F D D T P I A K A L Y R N W R E C K F V E D E G D V V F Y

          1300 1305
          AAGCATTAA
          AAGCATTAA
          AAGCATTAA
          K H
          K H
          K H
```

Figure S5
